# Biological and Structural Characterization of a Host-Adapting Amino Acid in Influenza Virus

**DOI:** 10.1371/journal.ppat.1001034

**Published:** 2010-08-05

**Authors:** Shinya Yamada, Masato Hatta, Bart L. Staker, Shinji Watanabe, Masaki Imai, Kyoko Shinya, Yuko Sakai-Tagawa, Mutsumi Ito, Makoto Ozawa, Tokiko Watanabe, Saori Sakabe, Chengjun Li, Jin Hyun Kim, Peter J. Myler, Isabelle Phan, Amy Raymond, Eric Smith, Robin Stacy, Chairul A. Nidom, Simon M. Lank, Roger W. Wiseman, Benjamin N. Bimber, David H. O'Connor, Gabriele Neumann, Lance J. Stewart, Yoshihiro Kawaoka

**Affiliations:** 1 Division of Virology, Department of Microbiology and Immunology, Institute of Medical Science, University of Tokyo, Tokyo, Japan; 2 Influenza Research Institute, Department of Pathobiological Sciences, School of Veterinary Medicine, University of Wisconsin-Madison, Madison, Wisconsin, United States of America; 3 Emerald BioStructures, Inc., Bainbridge Island, Washington, United States of America; 4 Seattle Structural Genomics Center for Infectious Disease, Washington, United States of America; 5 Department of Microbiology and Infectious Diseases, Kobe University, Hyogo, Japan; 6 Department of Special Pathogens, International Research Center for Infectious Diseases, Institute of Medical Science, University of Tokyo, Tokyo, Japan; 7 ERATO Infection-Induced Host Responses Project, Saitama, Japan; 8 Seattle Biomedical Research Institute, Seattle, Washington, United States of America; 9 Departments of Global Health and Medical Education & Biomedical Informatics, University of Washington, Seattle, Washington, United States of America; 10 Faculty of Veterinary Medicine, Tropical Disease Centre, Airlangga University, Surabaya, Indonesia; 11 Collaborating Research Center-Emerging and Reemerging Infectious Diseases, Tropical Disease Centre, Airlangga University, Surabaya, Indonesia; 12 Wisconsin National Primate Research Center, University of Wisconsin-Madison, Madison, Wisconsin, United States of America; 13 Department of Pathology and Laboratory Medicine, University of Wisconsin-Madison, Madison, Wisconsin, United States of America; 14 Creative Research Initiative, Sousei, Hokkaido University, Sapporo, Japan; University of Maryland, United States of America

## Abstract

Two amino acids (lysine at position 627 or asparagine at position 701) in the polymerase subunit PB2 protein are considered critical for the adaptation of avian influenza A viruses to mammals. However, the recently emerged pandemic H1N1 viruses lack these amino acids. Here, we report that a basic amino acid at position 591 of PB2 can compensate for the lack of lysine at position 627 and confers efficient viral replication to pandemic H1N1 viruses in mammals. Moreover, a basic amino acid at position 591 of PB2 substantially increased the lethality of an avian H5N1 virus in mice. We also present the X-ray crystallographic structure of the C-terminus of a pandemic H1N1 virus PB2 protein. Arginine at position 591 fills the cleft found in H5N1 PB2 proteins in this area, resulting in differences in surface shape and charge for H1N1 PB2 proteins. These differences may affect the protein's interaction with viral and/or cellular factors, and hence its ability to support virus replication in mammals.

## Author Summary

Influenza viruses that originate from avian species likely have to acquire adapting amino acid changes to replicate efficiently in mammals. Two amino acid changes in the polymerase PB2 protein—a glutamic acid to lysine change at position 627 or an aspartic acid to asparagine change at position 701—are known to allow influenza viruses of avian origin to replicate efficiently in mammals. Interestingly, the pandemic H1N1 viruses (which possess an avian-like PB2 gene) do not encode the ‘human-type’ amino acids PB2-627K and PB2-701N. Here, we report that a basic amino acid at position 591 of PB2 can compensate for the lack of PB2-627K and allows efficient replication of highly pathogenic H5N1 and pandemic H1N1 viruses in mammalian species. We also present the X-ray crystal structure of the C-terminal portion of a pandemic H1N1 PB2 protein. The basic amino acid at position 591 fills a distinctive cleft found in the PB2 proteins of H5N1 viruses. We also speculate on the biological significance of the altered surface of the H1N1 PB2 protein.

## Introduction

Influenza viruses pose an ongoing threat to human health, as underscored by the current H1N1 influenza pandemic and the sporadic transmission of highly pathogenic avian H5N1 influenza viruses to humans [1], [2]. Viral determinants of virulence and transmissibility are still poorly understood, although lysine at position 627 of the polymerase subunit PB2 (PB2-627K) is now known to be important for avian influenza virus adaptation to mammals [3], [4]. Most avian influenza viruses (with the exception of the Qinghai Lake-lineage of H5N1 viruses) possess glutamic acid at this position (PB2-627E), whereas most human influenza viruses (with the notable exception of the current pandemic H1N1 viruses) have lysine (PB2-627K). In addition, replacement of the aspartic acid at position 701 of PB2 (PB2-701D) found in most avian influenza viruses with asparagine (PB2-701N) conferred high pathogenicity to an H5N1 influenza virus in mice [5]. These mammalian-type amino acids (*i.e.*, PB2-627K and PB2-701N) are found in some H5N1 (http://www.flu.lanl.gov) or H7N7 [6] influenza viruses isolated from humans, are selected during replication of H5N1 viruses in humans [7], and facilitate virus transmission in ferret [8] and guinea pig models [9]. Collectively, these results have led to the concept that PB2-627K or PB2-701N are critical for efficient influenza virus replication in mammalian species. Nevertheless, the pandemic H1N1 viruses and some H5N1 influenza viruses isolated from humans do not possess these amino acids. Here, we sought to identify additional amino acid changes that facilitate virus adaptation in mammalian species.

## Results/Discussion

### Lysine at position 591 of the PB2 protein confers efficient replication to an H5N1 influenza virus in mammals

**Figure 1:**
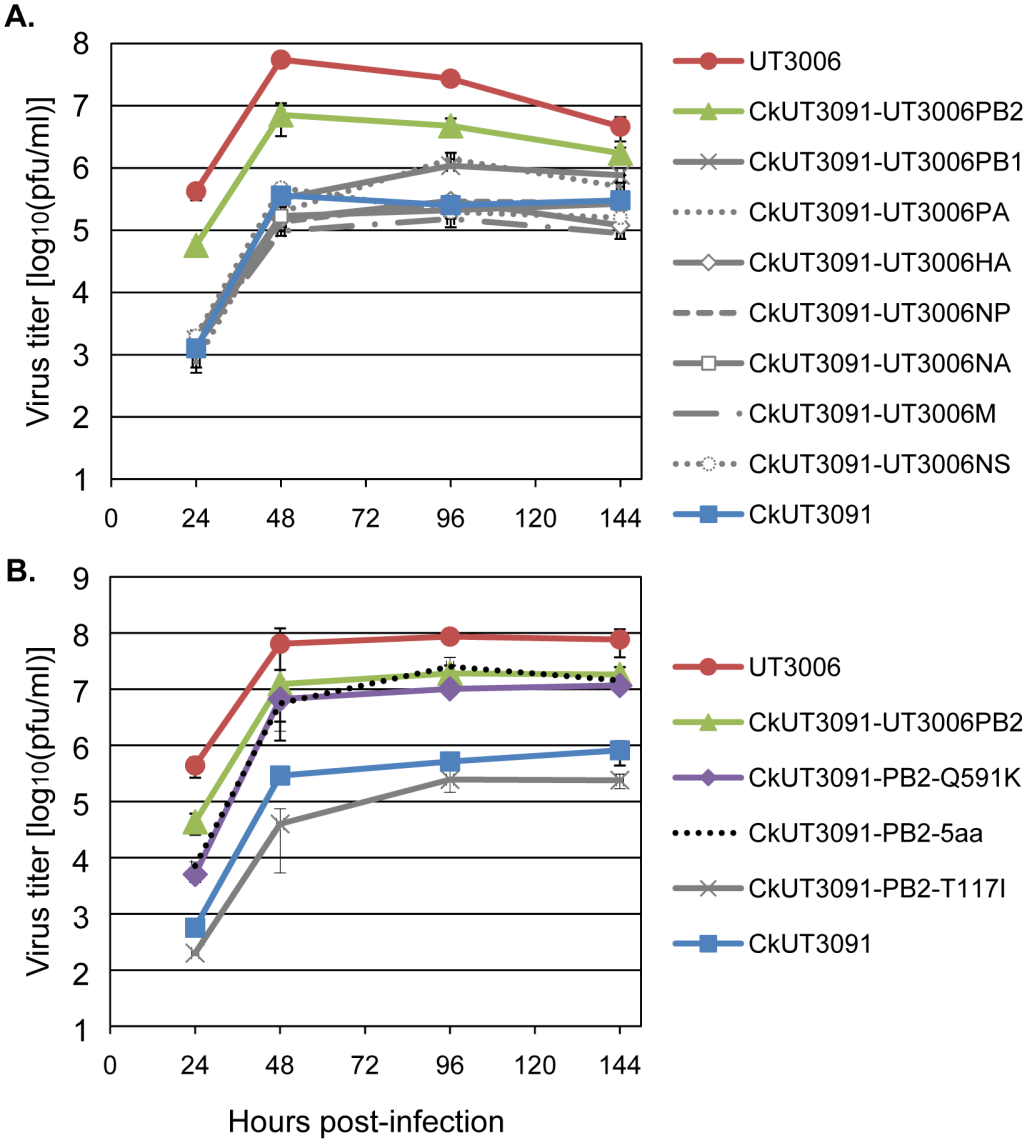
Amino acid PB2-591 is important for H5N1 virus replication in mammalian cells. **A**. We inoculated the human UT3006 virus, the avian CkUT3091 virus, or CkUT3091-based reassortant viruses possessing single genes of UT3006 (CkUT3091-UT3006PB2, -UT3006PB1, -UT3006PA, -UT3006HA, -UT3006NP, -UT3006NA, -UT3006M, and -UT3006NS) into normal human bronchioepithelial (NHBE) cells at a multiplicity of infection of 0.0005 and cultured infected cells at 37°C. The culture supernatants were harvested at the indicated times and subjected to plaque assays in NHBE cells to determine virus titers. **B**. Growth curves in NHBE cells of UT3006, CkUT3091, and CkUT3091 variants possessing UT3006-derived PB2 substitutions at position 117 (PB2-T117I), 591 (PB2-Q591K), or at all five positions indicated in the text (PB2-5aa). The CkUT3091 virus possessing the UT3006-PB2 gene (CkUT3091-UT3006PB2, see Fig. 1A) is shown again for comparison. Experiments to determine viral growth kinetics were carried out twice, with three explicates each. Shown are data from one experiment.

An H5N1 influenza virus isolated from an infected person in Indonesia in 2005 (A/Indonesia/UT3006/05, UT3006) replicated more efficiently than an avian H5N1 virus (A/chicken/Indonesia/UT3091/05 virus; CkUT3091) in normal human bronchioepithelial (NHBE) cells (Fig. 1), despite having avian-type amino acids at PB2-627 and PB2-701. In addition, the mouse lethal dose 50 (MLD_50_) of UT3006 is 180 plaque-forming units (PFU), indicating appreciable virulence in mice. Reverse genetics approaches demonstrated a critical role of the UT3006 PB2 segment in facilitating more efficient growth of UT3006-CkUT3091 reassortants in NHBE cells (Fig. 1A). Although the UT3006 and CkUT3091 PB2 proteins both encode the avian-type amino acids at positions 627 and 701, they differ by nine other amino acids (62-R/K, 117-T/I, 288-R/Q, 344-M/V, 524-T/I, 526-K/R, 591-Q/K, 676-K/T, 756-T/M; where the first amino acid indicates the residue found in CkUT3091 and the second indicates that found in UT3006). Five of these changes (at positions 62, 117, 524, 526, and 591) are not commonly found in avian influenza viruses, suggesting that they may play a role in mammalian adaptation. Introduction of the UT3006 sequence at these five sites appreciably increased the replicative ability of CkUT3091 in NHBE cells (CkUT3091-5aa, Fig. 1B), although virus titers did not reach the level of UT3006 virus. The respective changes were tested individually for their ability to facilitate avian influenza virus replication in human cells and introduction of the PB2-Q591K change appreciably enhanced the growth properties of CkUT3091 in NHBE cells (Fig. 1B). In contrast, the CkUT3091 variant possessing the PB2-T117I mutant was comparable in its growth to the parental CkUT3091 virus (Fig. 1B). The mutants containing PB2-R62K, PB2-T524I, or PB2-K526R changes were not viable, suggesting that these residues require compensatory changes at other sites. These findings suggest that, in the absence of PB2-627K or 701N, PB2-591K is a key amino acid for efficient influenza virus replication in human cells. Hence, replacement of the highly conserved glutamine at position 591 with lysine may facilitate mammalian adaptation in the context of an avian-type PB2 protein.

**Figure 2:**
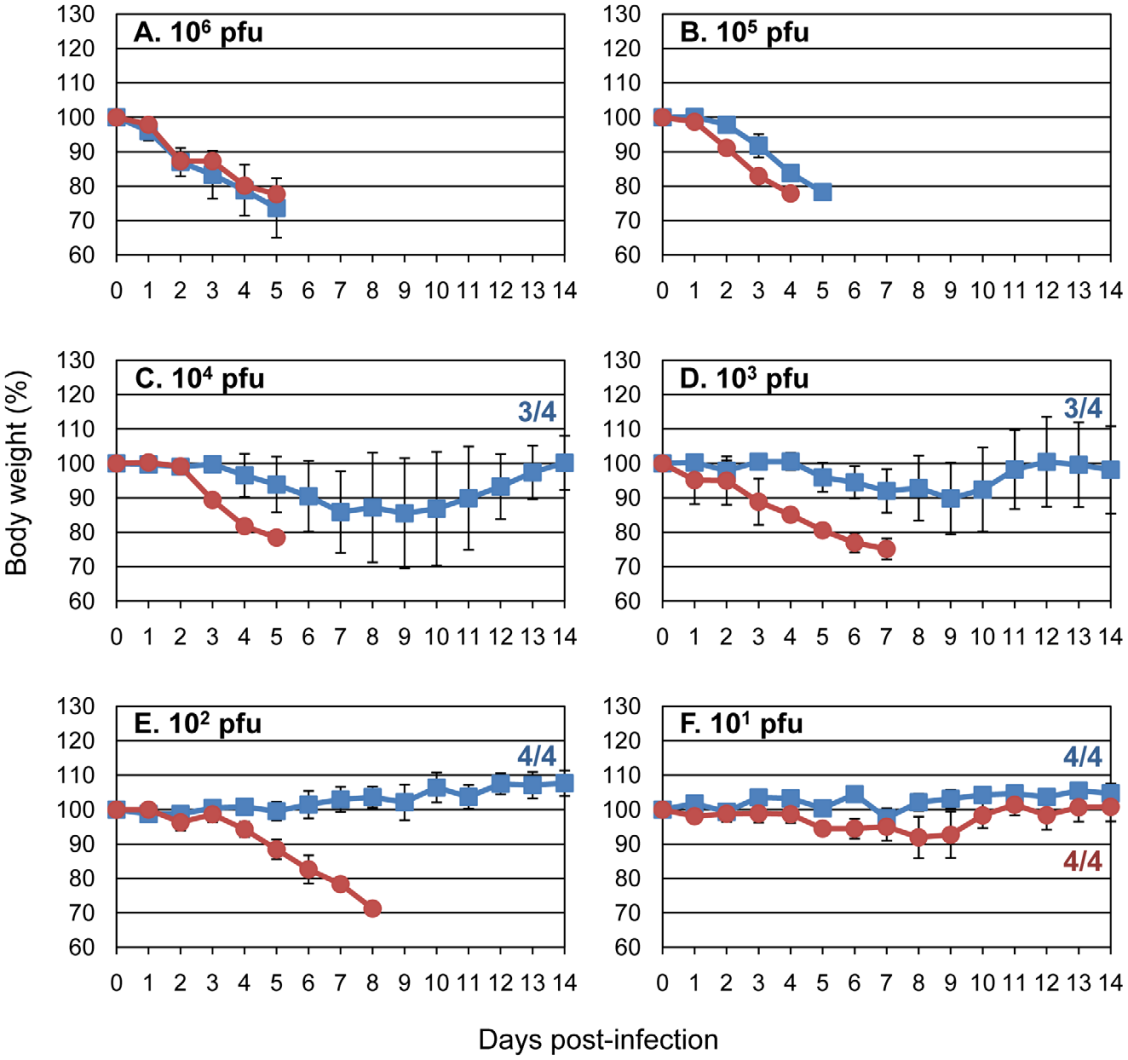
Amino acid PB2-591K increases the virulence of an avian H5N1 virus in mice. Mice (4 per group) were inoculated with the indicated amounts of CkUT3091 virus (blue squares) or CkUT3091 virus possessing the PB2-Q591K substitution (red circles) and observed daily for changes in body weight. Animals that lost more than 25% of their pre-infection weight were euthanized. The numbers in the graphs indicate the numbers of surviving mice per group. **A**.–**F**. Infection of mice with 10^6^ pfu (**A**), 10^5^ pfu (**B**), 10^4^ pfu (**C**), 10^3^ pfu (**D**), 10^2^ pfu (**E**), or 10^1^ pfu (**F**) of virus.

Next, we tested CkUT3091 and its mutant possessing the PB2-Q591K substitution (CkUT3091-PB2-Q591K) for their virulence in mice. All mice infected intranasally with high doses of virus [10^6^ or 10^5^ PFU of virus] lost more than 25% of their pre-infection weight and had to be euthanized on day 4 or 5 post-infection (Fig. 2A and B). At lower infection doses (10^2^–10^4^ PFU), most animals infected with CkUT3091 virus survived (Figs. 2C–E), while all mice infected with CkUT3091-PB2-Q591K had to be euthanized. At the lowest doses tested (10^1^ PFU), infected animals did not experience significant weight loss and survived (Fig. 2F). The MLD_50_ was 10^4.3^ PFU for CkUT3091 and 50 PFU for the mutant possessing PB2-Q591K (CkUT3091-PB2-Q591K). These findings demonstrated that the glutamine-to-lysine mutation at position 591 of PB2 increased the virulence of an avian H5N1 influenza virus in mice.

### Lysine at position 591 of the PB2 protein is selected during H5N1 virus replication in ostrich cells

We previously reported that the human-adapting amino acids PB2-627K and PB2-701N are not only selected during replication of avian influenza viruses in mammalian cells, but also during replication of avian H5N1 viruses in cultured primary ostrich cells or ostriches [10]. Although not fully understood, the selection processes in ostriches thus appear to mimic those in mammalian cells. Since the significance of PB2-591 had not been recognized at the time, we focused our previous analysis on PB2-627 and PB2-701. Here, we therefore reexamined all amino acid changes acquired during replication of A/duck/Vietnam/5001/04 (H5N1) or A/duck/Vietnam/NCVD-18/03 (H5N1) in ostrich cells *in vitro* or *in vivo*. PB2-591K was not detected after replication of A/duck/Vietnam/NCVD-18/03 in an infected ostrich, but 12 of 73 PB2 molecular clones derived from viruses isolated from the trachea of an ostrich infected with A/duck/Vietnam/5001/04 (originally possessing PB2-591Q) had acquired the PB2-591K substitution (Table 1). Interestingly, five passages of A/duck/Vietnam/5001/04 in ostrich embryonic cells also yielded one clone (out of 16) with a PB2-Q591R mutation, as found in pandemic H1N1 viruses (see below). Comparable to mammalian cells, the replication of avian H5N1 influenza viruses in ostriches thus resulted in the selection of a basic amino acid at position 591; it is interesting to note that both PB2-591K (as found in some mammalian-adapted H5N1 viruses) and PB2-591R (as found in pandemic H1N1 viruses) were detected. The observed differences in adaptation to ostriches between the two duck viruses may result from differences in their genetic composition. The selection of a basic amino acid at PB2-591 in ostrich cells that seem to mimic the selective pressure in mammals provided further support for the role of PB2-591 in avian influenza virus adaptation to mammalian species.

**Table 1:** Mammalian-type amino acids in the PB2 protein of ostrich isolates.

Virus^a^	No. of clones	PB2 amino acid^b^ at position:		
		591	627	701
Inoculum	50/50	Q	E	D
Brain isolate	35/35	Q	***K***	D
Trachea isolate	61/73	Q	***K***	D
	12/73	***K***	E	D
Lung isolate	33/57	Q	E	D
	24/57	Q	***K***	D

a We intratracheally inoculated A/duck/Vietnam/5001/04 (H5N1) into a 3-week-old ostrich and isolated virus from trachea, lungs, and brain on day 3 post-infection (for more details, see [10]). Viral RNA was isolated, and the PB2 gene was amplified by RT-PCR and cloned. We then sequenced the indicated numbers of PB2 clones. Results for PB2-627 and PB2-701 were already shown in Shinya *et al.* [10]. We here expanded our analysis to include PB2-591 in this study.

b Mammalian-type amino acids are shown in bold and italics.

All 12 ostrich PB2 clones possessing the PB2-591K mutation encoded PB2-627E (*i.e.*, the wild-type, avian-type amino acid; Table 1). Conversely, the remaining 61 clones acquired the PB2-627K (mammalian-type) mutation but encoded the wild-type amino acid at position 591 (PB2-591Q) (Table 1). These findings from our *in vivo* selection study are consistent with three published ostrich sequences (Supplementary Figure S1) that show the PB2-591Q/627K or PB2-591K/627E combinations, but not mutations at both positions (*i.e.*, PB2-591K/627K). Hence, PB2-591K appears to be selected in combination with PB2-627E, but not in combination with PB2-627K, suggesting that PB2-591K may functionally compensate for the lack of a positive charge at position 627.

### Role of PB2 amino acids 591, 627, and 701 in pandemic H1N1 viruses

Our data indicated that a basic amino acid at PB2-591 can compensate for PB2-627K in mammalian adaptation; interestingly, these two residues are positioned near each other on the surface of PB2 [11], [12]. These findings offered an explanation for the efficient replication of the newly emerged pandemic H1N1 viruses in humans: although these viruses lack mammalian-type amino acids at position 627 and 701, they encode a basic amino acid (i.e., arginine) at position 591 (PB2-591R). In fact, a recent study demonstrated that PB2-590S and PB2-591R are important for efficient polymerase activity in an *in vitro* assay, and for efficient replication of a mixed human/avian influenza virus (with the polymerase and nucleoproteins of avian virus origin) in human cells [13]. Importantly, the additional introduction of PB2-627K did not increase the replicative ability of the mutant virus further [13], consistent with our finding that the combination of basic amino acids at positions 591 and 627 is not selected for *in vivo*. However, these studies were not carried out with authentic pandemic H1N1 virus and therefore leave in question the biological significance of PB2-591R for the efficient replication of pandemic H1N1 viruses in humans.

To test the significance of specific PB2 amino acid changes in the background of authentic pandemic H1N1 viruses in animal models, we first generated the following PB2 protein variants that were all based on A/California/04/09 (Cal04), an early pandemic H1N1 virus that we previously characterized *in vitro* and in several animal models [14]: (*i*) wild-type PB2, (*ii*) PB2-627K (mammalian-type) instead of PB2-627E (avian-type), (*iii*) PB2-701N (mammalian-type) instead of PB2-701D (avian-type), or (*iv*) PB2-591Q (consensus amino acid at this position) instead of PB2-591R (found in pandemic H1N1 viruses). These variants were assessed for their polymerase activity in minireplicon assays, essentially as described [15]. Replacement of PB2-591R with PB2-591Q reduced the polymerase activity (Supplementary Figure S2), further indicating a critical role of PB2-591R in efficient pandemic H1N1 virus replication. Replacement of PB2-627E with PB2-627K, or of PB2-701D with PB2-701N, had mild or no effects. Herfst *et al.* [16] recently reported increased polymerase activity in minireplicon assays for pandemic H1N1 variants possessing the PB2-627K or PB2-701N mutations, but used different pandemic H1N1 virus isolates.

**Figure 3:**
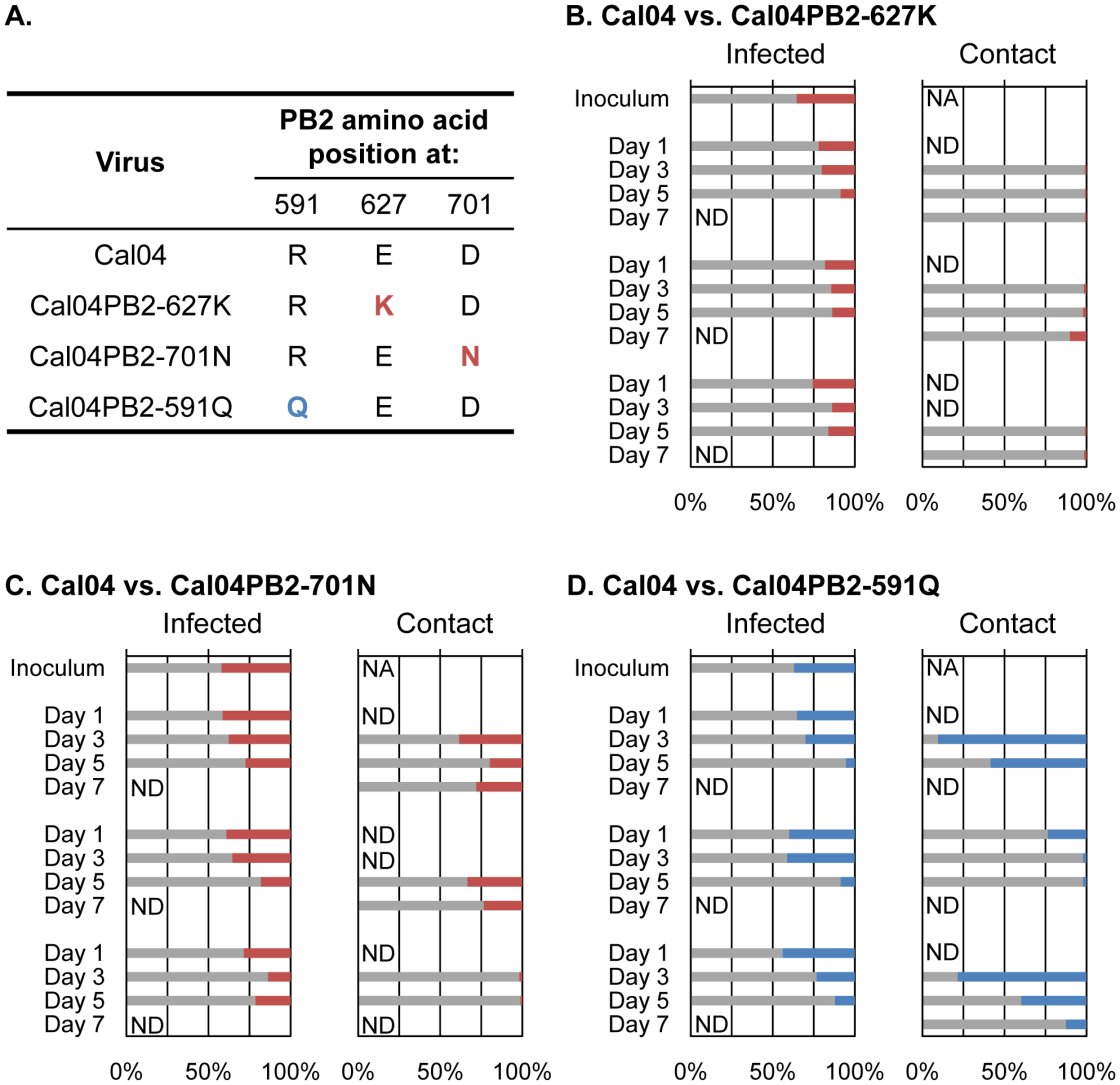
Competitive transmission studies in ferrets for wild-type and mutant pandemic H1N1 viruses. **A**. Overview of PB2 amino acids at positions 591, 627, and 701. Shown are A/California/04/09 (H1N1; Cal04) and its mutants tested in competitive transmission studies. **B**.–**D**. The indicated pairs of viruses were mixed at a 1∶1 ratio and three ferrets (one per cage) were infected intranasally with 10^6^ plaque-forming units of virus mix. One day later, one ferret each was placed in a cage adjacent to an infected ferret (‘contact’ ferret); that is, for each set of viruses, three pairs of infected/contact ferrets were tested. Virus populations in the nasal washes of infected and contact ferrets were assessed at the indicated time points. Grey bars; percent wild-type PB2 molecules. Colored bars; percent mutant PB2 molecules. NA, not applicable; ND, not determined (nasal wash samples with virus titers of less than 10^2^ plaque-forming units per ml were not processed for sequence analysis). Competition studies were carried out for Cal04 (PB2-591R/627E/701D) and Cal04PB2-627K (PB2-591R/627K/701D) (**B**), Cal04 (PB2-591R/627E/701D) and Cal04PB2-701N (PB2-591R/627E/701N) (**C**), and for Cal04 (PB2-591R/627E/701D) and Cal04-PB2-591Q (PB2-591Q/627E/701D) (**D**).

Next, the PB2 mutations described above were introduced into the genetic background of Cal04 virus, using reverse genetics. Growth curves in Madin-Darby canine kidney (MDCK) cells revealed no significant differences (Supplementary Figure S3), as has been reported by others for the PB2-627K and PB2-701N variants [16], [17]. Intranasal inoculation of BALB/c mice with 10^4^ or 10^5^ PFU of virus resulted in increased weight loss for the PB2-627K and PB2-701N variants (Supplementary Figure S4); however, virus titers in the lungs and nasal turbinates on days 3 and 6 post-infection were comparable for all variants tested (Supplementary Table S1). Similar findings (i.e., weight loss in mice, but no increase in virus titers) were recently reported by others for pandemic H1N1 viruses possessing PB2-627K or PB2-701N [16], [17]. The effects of mutations in PB2 on pandemic H1N1 viruses were further tested in competitive transmission studies in ferrets, an established animal model in influenza virus research; in addition, pandemic H1N1 viruses are known to efficiently transmit in ferrets [14], [18], [19]. Wild-type Cal04 (PB2-591R/627E/701D) and its PB2 variants (PB2-591R/627K/701D, PB2-591R/627E/701N, or PB2-591Q/627E/701D) were mixed at a 1∶1 ratio based on plaque-forming units; however, deep sequencing revealed a slight excess of wild-type Cal04 PB2 vRNA over mutant PB2 vRNAs (Fig. 3B–D). Three ferrets (one per cage) were infected intranasally with virus mix. One day later, one ferret each was placed in a cage adjacent to an infected ferret (‘contact’ ferret) to monitor virus transmission by aerosols. At the indicated time points, nasal wash samples were assessed for the relative ratios of the two viruses (Figs. 3B–D; and Supplementary Table S2). In both infected and contact ferrets, mutations at position PB2-627 (PB2-591R/627K/701D) or PB2-701 (PB2-591R/627E/701N) did not provide a replicative advantage to the respective viruses when compared to wild-type Cal04 virus (PB2-591R/627E/701D). This is consistent with recent findings that E627K or D701N mutations did not affect the virulence or transmissibility of pandemic H1N1 viruses in ferrets [16]. However, in competition studies between wild-type Cal04 virus and a mutant virus possessing PB2-591Q (PB2-591Q/627E/701D), the relative amounts of Cal04 virus increased over time in infected animals (Fig. 3D), indicating that in the background of PB2-627E/701D, PB2-591R provides a replicative advantage over PB2-591Q (the amino acid commonly found at this position). Interestingly, two out of three contact animals showed enrichment of the PB2-591Q mutant virus on day 3 after co-housing; thus, although further studies with larger numbers of animals are needed, we speculate that PB2-591Q might enhance transmission, but not replication in the new host. Collectively, our findings supported the hypothesis that PB2 possessing 591R supports efficient viral replication in mammals, so that there is no strong selective pressure to acquire the mammalian-type amino acids at position 627 and 701.

Based on our experimental data and the evaluation of published influenza virus sequences, we suggest the following scenario: efficient replication in mammalian species can be conferred by PB2-591Q/627K [found in some avian H5N1 viruses that acquired the ability to replicate in mammalian cells [7] (http://www.flu.lanl.gov), and found in human influenza viruses], PB2-591K/627E [found in some avian H5N1 viruses that acquired the ability to replicate in mammalian cells, (http://www.flu.lanl.gov), and identified in this study], or PB2-591R/627E (found in pandemic H1N1 viruses). However, the combination of PB2-591R/K and PB2-627K does not seem to further enhance replication in mammalian cells (Figs. 3B and C). Currently, it is not clear why PB2-591R is found in pandemic H1N1 viruses, while PB2-591K is selected in avian H5N1 viruses. In addition, PB2-701N also confers to H5N1 viruses the ability to replicate in mammalian cells in the absence of PB2-627E [5]. Moreover, other amino acids changes in PB2 may confer efficient replication in mammalian species as well (for example, [20], [21]).

### Crystal structure of the C-terminal portion of PB2 from a 2009 H1N1 pandemic virus

**Figure 4:**
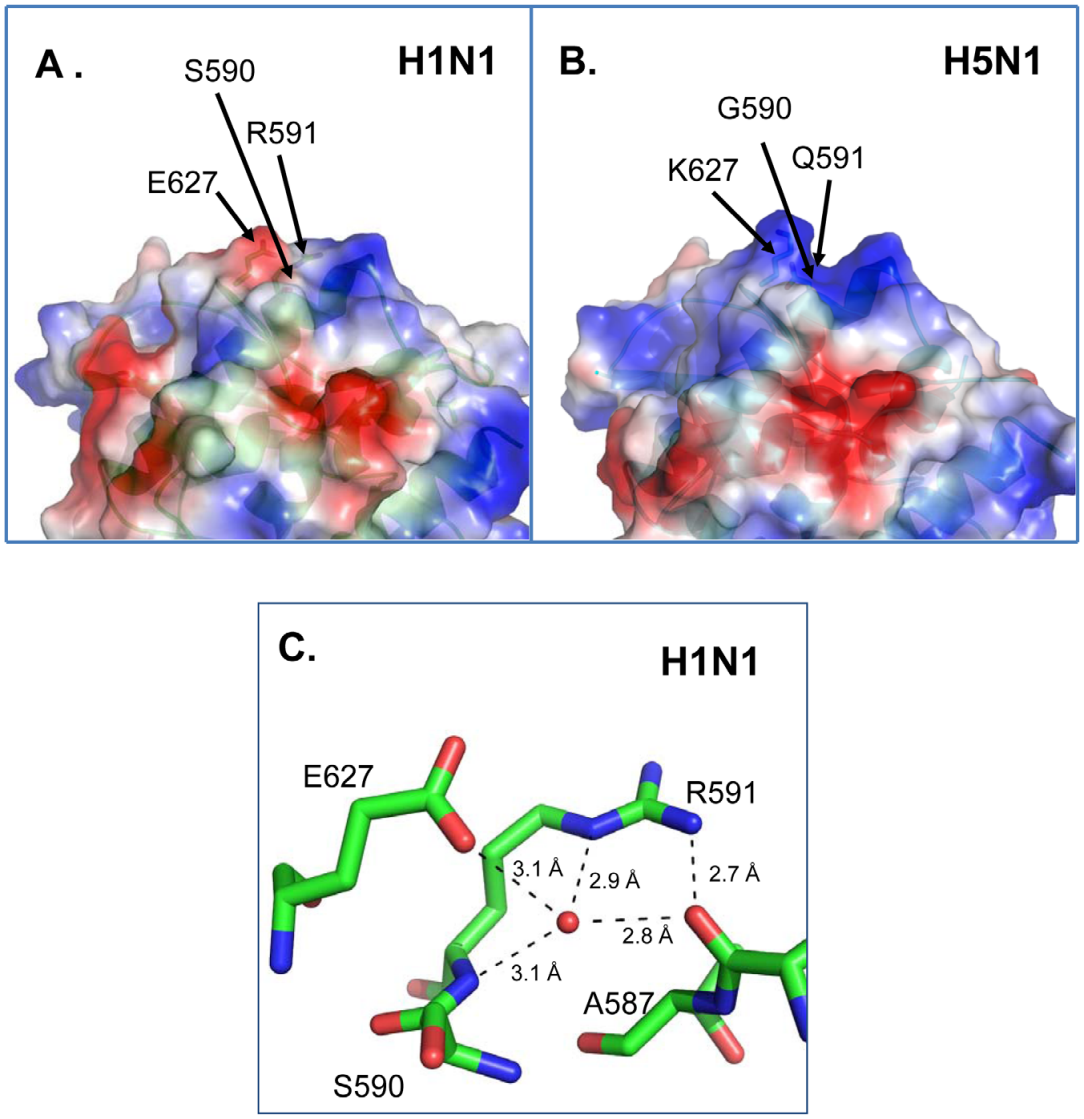
X-ray crystal structures of pandemic H1N1 and avian H5N1 PB2 proteins. Shown are the X-ray crystal structures of a pandemic H1N1 (PB2-591R/627E) and an H5N1 PB2 protein encoding PB2-627K (PB2-591Q/627K). **A**. The surface of H1N1-PB2 in the region of PB2-591R and PB2-627E (PDBID:3KHW). Color of surface is mapped by qualitative vacuum electrostatics generated by PYMOL (27). Blue = relative positive charge, Red = relative negative charge. Residue S590, R591, and E627 are shown as stick representations underneath the surface contour and are identified with black arrows. The side chain of R591 intrudes into the cleft shown in Fig. 4B. **B**. The surface of H5N1-PB2 in the region of PB2-591Q and PB2-627K (PDBID:3KC6) shows a distinct cleft in comparison to the surface of H1N1 PB2-591R and PB2-627E (PDBID:3KHW). **C**. Close-up view of the interactions of H1N1 PB2-591R with neighbouring residues. H1N1-PB2 residues A587, S590, R591, and E627 are shown in sticks model, green = carbon, red = oxygen, blue = nitrogen. The conformation of H1N1 PB2-591R (PDBID:3KHW) is shown oriented away from PB2-627E making hydrogen bonding and water-mediated hydrogen bonding interactions to the carbonyl oxygen of residue PB2-587A. PB2-591R fills the cleft shown in Figure 4B. Residue S590 sits underneath E627 and blocks the conformational freedom of E627.

Previous X-ray crystallographic studies have demonstrated the close proximity of residues 591 and 627 on the surface of PB2 [11], [12]. This has lead to the suggestion (based on modeling) that PB2-591R neutralizes the PB2-627E in pandemic H1N1 viruses, and thereby partially restores the positively charged surface of the 627 domain of PB2 [13]. To address this critical question, we present here the high-resolution crystal structure of the C-terminal domain of PB2 from a 2009 H1N1 pandemic virus (A/Mexico/InDRE4487/2009) (Fig. 4A); the PB2 proteins of A/Mexico/InDRE4487/2009 and Cal04 are identical in the region crystallized; i.e., amino acid residues 538–741. For comparison, we also present the crystal structure of the C-terminal portion of an H5N1 PB2 protein encoding PB2-627K (A/Vietnam/1203/04; Fig. 4B).

The PB2 protein of the pandemic H1N1 virus shows an altered polar surface when compared to the avian H5N1 PB2-627K protein. Interestingly, our structure of the pandemic H1N1 PB2 protein reveals that PB2-591R fills the cleft found in H5N1 PB2 proteins in this area (compare Fig. 4A and 4B). In particular, the side chains of R591 and S590 in the structure of H1N1 PB2 occupy what would otherwise be the positively charged cleft in the H5N1 PB2. PB2-591R is located 4 Ångstrom from PB2-627E, with the side chain of R591 rotated away from PB2-627E and participating in hydrogen-bonded and water-mediated hydrogen bond networks with the main chain carbonyl backbone between residues A587 and T588 (Fig. 4C), even though the two residues have space to assume rotomers that could form a neutralizing salt bridge (see Fig. 4C). The R591 residue in H1N1 PB2 thus affects both the shape and charges on the surface of the protein, which may affect its interaction with other viral and/or host factors.

A previous study [22] suggested that an inhibitory host factor may suppress the activity of avian-type polymerases (possessing PB2-627E), but not that of human-type polymerases (possessing PB2-627K). In this scenario, the positively charged residue (PB2-591R) that rotates away from PB2-627E may prevent the inhibitory host factor from interacting with PB2 by either charged repulsion or steric hindrance by filling the distinct cleft on the surface of the H5N1 PB2. Alternatively, the positive charge associated with PB2-627K or PB2-591R may facilitate interaction with a stimulatory host factor. Thus, efficient replication in mammalian cells may require a PB2 structure that prevents the action of the inhibitory host factor, or facilitates the action of the stimulatory host factor. Our finding that PB2-591Q/627K (found in human viruses) and PB2-591R/627E (found in pandemic H1N1 viruses) adopt different conformations (*i.e.* a positively charged, ‘cleft’ *vs.* a positively-charged residue that fills the cleft and is located next to a negatively-charged patch) probably favors the former possibility, since it is less likely that they would both enhance interaction with the same host protein, but the positive charge introduced by either change could easily disrupt interaction with an inhibitory host protein.

A serine at position 590 of PB2 is found in pandemic H1N1 viruses (but not consistently in older human H1N1 isolates), and this residue (PB2-590S) has been suggested to play a role in the enhanced replicative ability of pandemic H1N1 viruses in mammals [13]. Our results that a basic amino acid at PB2-591 provided a replicative advantage in mammals to avian H5N1 viruses (which possess a glycine at PB2-590), argue against a critical role of PB2-590S in the adaptation of H5N1 influenza viruses to mammals. Our H1N1 structure shows that while the side chain of PB2-590S is in close proximity (<4 Ångstroms) to 627E, the hydroxyl moiety points away from the glutamatic acid and does not participate in hydrogen bonds or polar interactions with the side chains of 591R or 627E. Furthermore, the side chain atoms of PB2-590S also protrude into the positively charged cleft and may provide a steric platform that reduces the conformational flexibility of neighboring residue 627E. These findings suggest that PB2-590S constrains any side chain at position 627. This constraint may be critical for PB2-627K which may need conformational freedom to reach its binding partner. The constraint by PB2-590S on PB2-627E may be less critical, and may in fact help to shield the negative charge.

Our study identifies a new marker (PB2-591R or K) for influenza virus adaptation in mammals that compensates for the lack of PB2-627K. This finding provides an explanation for the efficient replication of pandemic H1N1 viruses (which possess PB2-591R) in mammals. Our X-ray crystal structure of the C-terminal portion of a pandemic H1N1 PB2 protein reveals changes in surface shape and charge created by PB2-591R which may prevent an inhibitory host factor as suggested by Mehle & Doudna [22] from binding to PB2, hence allowing efficient influenza virus replication. Although a few human H1N1 isolates have now been found to possess PB2-627K (http://www.promedmail.org/pls/otn/f?p=2400:1001:19224::NO::F2400_P1001_BACK_PAGE,F2400_P1001_PUB_MAIL_ID:1010,79432), this mutation did not appear to increase the severity of disease, and does not seem to spread in human populations, consistent with our conclusion that the PB2-627K mutation does not provide a significant replicative advantage to pandemic H1N1 viruses. Based on findings with H5N1 influenza viruses, it was feared that the introduction of PB2-627K into pandemic H1N1 viruses could increase the pathogenicity of the pandemic viruses. However, our data and recent findings by others [13] indicate that PB2-627K does not provide a replicative advantage in the background of a PB2 protein possessing a basic amino acid at PB2-591. From a public health perspective, the notion that the introduction of PB2-627K into pandemic H1N1 viruses is rare and unlikely to create a more pathogenic variant is thus reassuring.

## Materials and Methods

### Viruses and cells

Human embryonic kidney (293 or 293T) cells were maintained in Dulbecco's modified essential medium (DMEM) containing 10% fetal calf serum and antibiotics. Madin-Darby canine kidney (MDCK) cells were maintained in Eagle's minimal essential medium (MEM) containing 5% newborn calf serum. Normal human bronchioepithelial cells (NHBE) were obtained from Lonza (Walkersville, MD) and maintained in serum-free and hormone-supplemented growth medium according to the manufacturer's instructions. All cells were incubated at 37°C with 5% CO_2_.

All influenza viruses used in this study were amplified in MDCK cells. All viruses were stored at −80°C until their use in experiments. The titers of stock viruses were determined by plaque assays in MDCK cells (for H1N1 viruses) or NHBE cells (for H5N1 viruses). All experiments with H5N1 viruses were performed in enhanced biosafety level 3 (BSL3) containment laboratories at the University of Wisconsin-Madison, which are USDA approved for such use by the CDC and the U.S. Department of Agriculture, or in BSL3 containment laboratories at the University of Tokyo (Tokyo, Japan), which are approved for such use by the Ministry of Agriculture, Forestry and Fisheries, Japan.

### Construction of plasmids and generation of wild-type and mutant influenza viruses

The cDNAs of the A/chicken/Indonesia/UT3091/05 (H5N1; CkUT3091), A/Indonesia/UT3006/05 (H5N1; UT3006), and A/California/04/09 (H1N1; Cal04) viruses were synthesized by reverse transcription as previously described [23], [24]. Viruses were amplified and titrated as described above. All viruses were sequenced to ensure the absence of unwanted mutations.

Mutations in the PB2 genes of CkUT3091 or Cal04 viruses were generated by PCR amplification of the respective RNA polymerase I PB2 construct with primers possessing the desired mutations (primer sequences will be provided upon request). All constructs were sequenced to ensure the absence of unwanted mutations.

### Minireplicon assays

To assess the viral polymerase activity, we performed luciferase activity-based minireplicon assay as described previously [15] using the following plasmids; expression plasmids for Cal04-PB2, -PB1, -PA, and -NP (0.2 µg each), pPolI-WNA-Fluc (0.02 µg), which express NA gene encoding firefly luciferase gene, and pGL4.74[hRluc/TK] (Promega, 0.02 µg). The luciferase activity in the plasmid-transfected 293 cells was measured by Dual-Luciferase Reporter Assay System (Promega) at 24 h post-transfection.

### Growth curves in NHBE or MDCK cells

NHBE cells (Lonza) were infected with the indicated viruses at a multiplicity of infection (m.o.i) of 0.0005. One hour later, the viral inoculum was removed and replaced with bronchial epithelial growth medium (SAGM; Cambrex) containing bovine pituitary extract (BPE; 30 µg/ml), hydrocortisone (0.5 µg/ml), human epidermal growth factor (hEGF; 0.5 ng/ml), epinephrine (0.5 µg/ml), transferrin (10 µg/ml), insulin (5 µg/ml), triiodothyronine (6.5 ng/ml), bovine serum albumin – fatty-acid free (BSA-FAF; 50 µg/ml), retinoic acid (RA; 0.1 ng/ml), gentamycin (30 µg/ml) and amphotericin B (15 ng/ml). Infected cells were incubated at 37°C and 5% CO_2_. At the indicated times post infection, the virus titers in the cell culture supernatant were determined by plaque assays in NHBE cells. For the determination of viral growth kinetics in MDCK cells, cells were infected at an m.o.i. of 0.001; at the indicated time points, virus titers were determined by plaque assays.

### Virulence in mice

Six-week-old female BALB/c mice (Japan SLC Inc., Shizuoka, Japan; The Jackson Laboratory, Bar Harbor, Maine, USA) were used in this study. Baseline body weights were measured prior to infection. Four mice per group were anesthetized with isoflurane and intranasally inoculated with 10^1^–10^6^ PFU (50 µl) of CkUT3001 or CkUT3091-PB2-Q591K, or with 10^3^ or 10^6^ PFU of Cal04 possessing wild-type or mutant PB2 protein. Body weight and survival were monitored daily for 14 days; mice with body weight loss of more than 25% of pre-infection values were euthanized. For virus titration in organs, mice were infected intranasally with 10^5^ PFU of virus and euthanized on days 3 and 6 postinfection.

### Ferret competition-transmission study

We used six-to-seven-month-old female ferrets (Triple F Farms, Sayre, PA), which were serologically negative by hemagglutination inhibition (HI) assay for currently circulating human influenza viruses (including pandemic H1N1 viruses). The animals were housed in adjacent transmission cages that prevent direct and indirect contact between animals but allow spread of influenza virus through the air. Three ferrets were intranasally inoculated with 10^6^ plaque-forming units (pfu; in 500 µl) of the mixture of Cal04 and mutant virus at a 1∶1 ratio (0.5×10^6^ pfu of Cal04 plus 0.5×10^6^ pfu of mutant virus). One day after infection, three naive ferrets were each placed in a cage adjacent to an inoculated ferret (contact ferrets). Hence, we tested three pairs of infected/contact ferrets for each virus. Nasal washes from ferrets were collected on days 1, 3, 5, and 7 post-infection or co-housing and titrated for virus titers in MDCK cells. Samples with greater than 1×10^2^ pfu/ml of virus were subjected to sequence analysis as described below. All animal experiments were carried out in accordance with institutional guidelines.

### Sequence analysis of viruses obtained from infected or contact ferrets

Viral RNA was extracted from nasal wash samples and twice from the inoculum (to verify the 1∶1 ratio of the two test viruses). The cDNAs were synthesized as previously described [23]. A panel of PCR primer pairs was used to generate 410 base pair amplicons of the PB2 region covering 591Q, 627K, and 701N of A/California/04/2009. All primers contained Roche/454 Titanium Amplicon adapter sequences and distinctive multiplier identifier (MID) tags engineered at their 5′ termini. After gel purification and normalization to 10^8^ molecules/µl, emulsion PCR was performed on four pools containing 14 MID-tagged amplicons each.

The resulting pools were pyrosequenced in individual regions of a 70×75 PicoTiterPlate with a 16-well gasket on a Roche/454 GS FLX instrument following protocols provided by the manufacturer (www.454.com). An average of 2221 sequence reads was obtained for each sample (range: 705–5102). All sequences from a single time point were aligned to the PB2 sequence of A/California/04/09 (Roche/454 AVA software). The frequencies of wild-type and mutant amino acids at residues 591, 627, and 701 were tabulated from the aligned sequences. To confirm the pyrosequencing results, independent PB2 sequences from 37 of the 42 samples were generated using alternative sets of PCR primers and pyrosequenced using the Roche/454 FLX chemistry.

### X-ray crystal structure determination of PB2 C-terminal domains

Codon engineered synthetic genes for the PB2 gene of influenza A virus strains A/Vietnam/1203/04 (H5N1) (AAT73550) and A/Mexico/InDRE4487/09 (H1N1) (ACQ73384) were designed in Gene Composer (Emerald BioSystems, Bainbridge Island, WA) using a combined *E. coli* and baculovirus codon usage table with a threshold setting of 0.2% and synthesized (DNA 2.0, Menlo Park, CA) [25], [26]. The C-terminal amino acid residues 538–741 from both strains were cloned into an engineered pET vector using the Polymerase Incomplete Primer Extension (PIPE) cloning technology [27]. Cloning, expression, and purification followed reported protocols from the Seattle Structural Genomics Center for Infectious Disease [28]. Constructs were expressed in vectors containing an N-terminal 6×His Smt fusion protein [29]. Site specific removal of the 11kDA 6×His Smt fusion partner was accomplished with 0.2 µg Ulp1 protease/mg of recombinant target [29], [30]. Final protein buffer composition following size exclusion chromatography (Superdex 75, GE Healthcare, Piscataway, NJ) was 25 mM Hepes/NaOH pH 7.2, 500 mM NaCl, 2 mM DTT, 5% glycerol. Protein crystallization trials were conducted by sitting drop vapor diffusion at 16°C in 96-well crystallization plates. Crystallization conditions for H5N1-PB2(538–741) were 5% octyl β-D-1-thioglucopyranoside (SBOG), 100 mM N-cyclohexyl-3-aminopropanesulfonic acid (CAPS) pH 10.5, 1.2 M sodium phosphate, 0.2 M lithium phosphate, 0.8 M potassium phosphate at protein concentration of 8 mg/ml. Crystallization conditions for H1N1-PB2(538–741) were 20% w/v PEG 3350, 200 mM ammonium citrate at protein concentration of 24 mg/ml. All crystal drop sizes used were 0.4 µl crystallant plus 0.4 µl protein. Crystals were harvested by transfer into drops containing crystallant plus either 25% glycerol or ethylene diol as cryoprotectant and flash frozen in liquid nitrogen. X-ray data were collected on a Rigaku (Rigaku Americas, The Woodlands, Texas) FR-E+ X-ray generator equipped with a Saturn-944 CCD detector. One degree images were collected for 30 seconds and scaled with HKL2000 [31]. Structures were determined by molecular replacement with the coordinates from PDBID 3CW4 using CCP4/PHASER [32]. Iterative refinement and modeling of coordinates was conducted using REFMAC [33] and COOT [34]. Final crystallographic refinement statistics are presented in Table 2. Structure figures were generated using PYMOL [35].

**Table 2:** X-ray crystallographic data collection and refinement statistics.

PDBID	3KHW	3KC6	3L56
**Protein**	PB2, 538–741	PB2, 538–741	PB2, 538–759
**Strain**	A/Mexico/INDRE4487/2009(H1N1)	A/Vietnam/1203/2004(H5N1)	A/Vietnam/1203/2004(H5N1)
**Data Collection**			
space group	P212121	P212121	P21
A	53.86	33.08	41.04
B	68.19	65.69	57.00
C	107.35	96.85	83.08
A	90	90	90
B	90	90	102.4
Γ	90	90	90
# molecules/asymmetric unit	2	1	2
Wavelength [Å]	1.54178	1.54178	1.54178
Resolution [Å](1)	50.0–2.08	50.0–2.08	50–2.30
	(2.12–2.08)	(2.12–2.08)	(2.34–2.30)
Redundancy	6.8 (4.6)	5.7 (4.4)	3.5 (2.2)
Unique	24405 (1121)	13176 (621)	16536 (678)
Completeness [%]	99.5 (93.4)	99.1 (96.6)	98.1 (82.0)
R_merge_ [%](2)	7.4 (20.9)	7.6 (27.7)	2.9 (4.6)
I/σI(3)	29.3 (5.6)	25.5 (5.3)	21.0 (18.5)
Mosaicity	0.6	0.4	0.8
**Refinement Statistics**			
No. of reflections	23670 (1626)	12197 (861)	16526 (940)
No. of non-hydrogen atoms	3362	1607	3270
Resolution range [Å]	50–2.10	50.0–2.10	50–2.30
	(2.15–2.10)	(2.15–2.10)	(2.36–2.30)
R_cryst_ (4)	19.7 (18.5)	19.9 (21.6)	18.0 (19.8)
R_free_ (5)	23.8 (25.1)	24.6 (34.3)	23.7 (27.3)
FreeR, # of reflections	5%, 1218 (88)	5%, 631 (49)	5%, 840 (53)
average Bfactor [Å^2^]	7.8	9.8	12.9
**Model Geometry**			
Bond length deviation [Å]	0.012	0.011	0.014
Bond angle deviation [°]	1.316	1.208	1.377

(1) Numbers in parenthesis represent highest resolution shell of data.

(2) *R*_merge_ = (|Σ*I*_hkl_−<*I*>|/(Σ*I*_hkl_), where the average intensity <*I*> is taken over all symmetry equivalent measurements and *I*_hkl_ is the measured intensity for any given reflection.

(3) *I/σI* is the mean reflection intensity divided by the estimated error.

(4) *R*_cryst_ = ∥*F*_o_|−|*F*_c_∥/|*F*_o_|, where *F*_o_ and *F*_c_ are the observed and calculated structure factor amplitudes, respectively.

(5) *R*_free_ is equivalent to *R*_cryst_ but calculated for 5% of the reflections chosen at random and omitted from the refinement process.

### Ethics

Our experiments in mice, ostriches, and ferrets followed the University of Tokyo's Regulations for Animal Care and Use and the University of Wisconsin-Madison's Animal Care and Use Protocol. They were approved by the Animal Experiment Committee of the Institute of Medical Science, the University of Tokyo (approval number: 19–29) and the Animal Care and Use Committee of the University of Wisconsin-Madison (protocol number V00806), which acknowledged and accepted both the legal and ethical responsibility for the animals, as specified in the Fundamental Guidelines for Proper Conduct of Animal Experiment and Related Activities in Academic Research Institutions under the jurisdiction of the Ministry of Education, Culture, Sports, Science and Technology, 2006 (Japan) and in the Animal Welfare Act and associated Animal Welfare Regulations and Public Health Service Policy (USA).

## Supporting Information

Figure S1 Alignment of PB2 protein sequences of influenza A viruses isolated from ostriches, emus, and rheas. Shown is the region covering amino acid positions 591, 627, and 701.
(0.44 MB TIF)

Figure S2 Polymerase activity of Cal04 PB2 variants *in vitro*. 293 cells were transfected with plasmids for the expression of a virus-like RNA encoding luciferase, and with plasmids encoding the Cal04 PB1, PA, NP, and wild-type or mutant PB2 proteins. Forty-eight hours later, luciferase activity was assessed.
(0.10 MB TIF)

Figure S3 Growth curves of wild-type and mutant Cal04 viruses in MDCK cells. Cells were infected with wild-type or mutant Cal04 viruses at an m.o.i. of 0.001. At the indicated times post-infection, the virus titers in the cell culture supernatant were assessed by plaque assays. Experiments were carried out in triplicate.
(0.10 MB TIF)

Figure S4 Virulence of wild-type and mutant Cal04 viruses in mice. BALB/c mice were infected with 10^5^ PFU (**A**) or 10^4^ PFU (**B**) of the indicated viruses and assessed daily for weight losses. For mice infected with the PB2-627K or PB2-701N variants, one mouse each had to be euthanized on day 5 or 6, respectively, due to body weight loss of more than 25% of the respective pre-infection body weight.
(0.17 MB TIF)

Table S1 Biological features of PB2 variants in mice
(0.03 MB DOC)

Table S2 Competitive transmission studies in ferrets for wild-type and mutant pandemic H1N1 viruses
(0.04 MB XLS)
