# Supplementary figures and images for: Biological and Structural Characterization of a Host-Adapting Amino Acid in Influenza Virus
Source: PLoS Pathog. 2010 Aug 5;6(8):e1001034. doi: 10.1371/journal.ppat.1001034 (PMC2916879; doi:10.1371/journal.ppat.1001034)

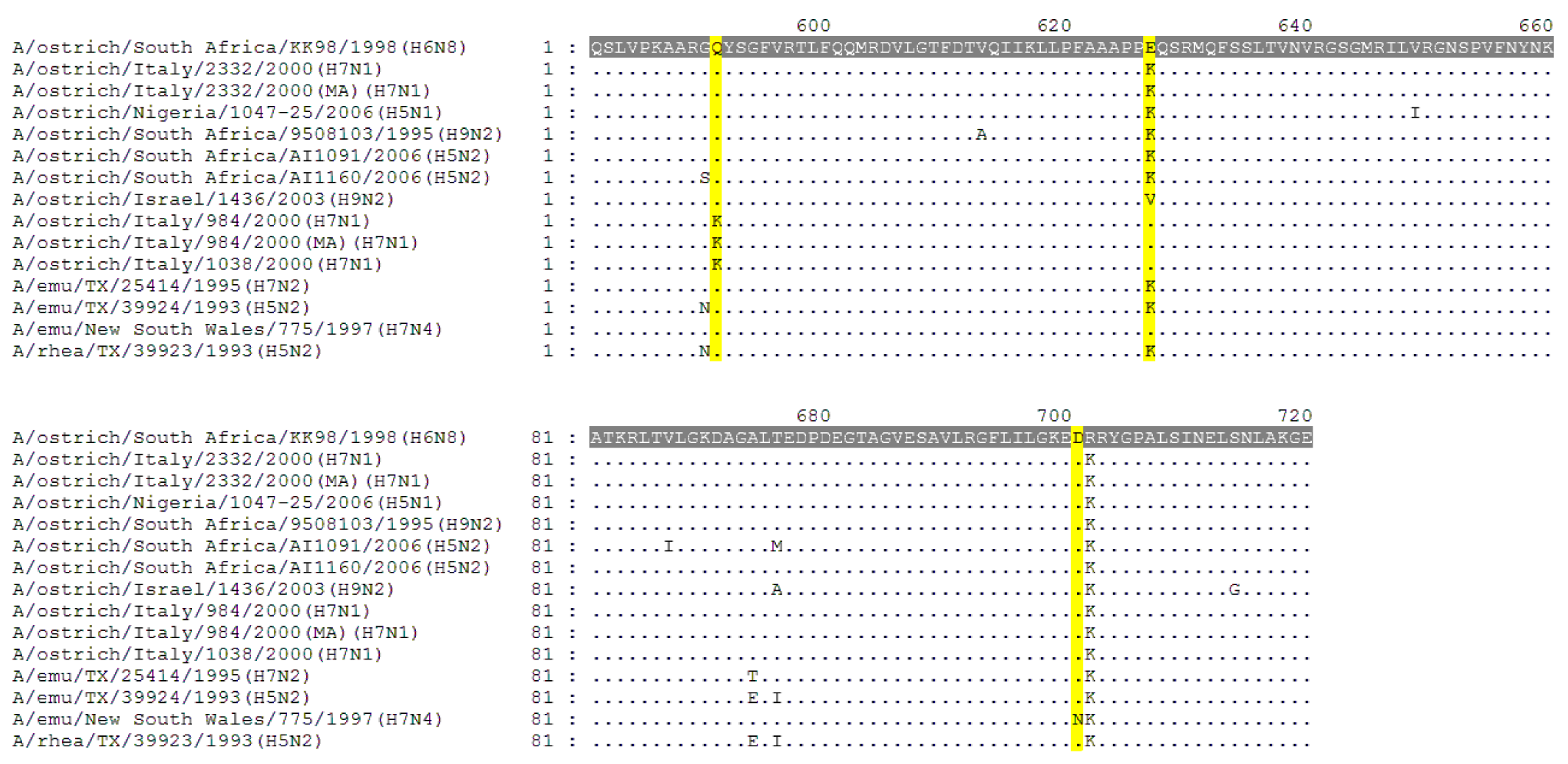

Supplement: Figure S1 — Alignment of PB2 protein sequences of influenza A viruses isolated from ostriches, emus, and rheas. Shown is the region covering amino acid positions 591, 627, and 701. (0.44 MB TIF) [file ppat.1001034.s001.tif]

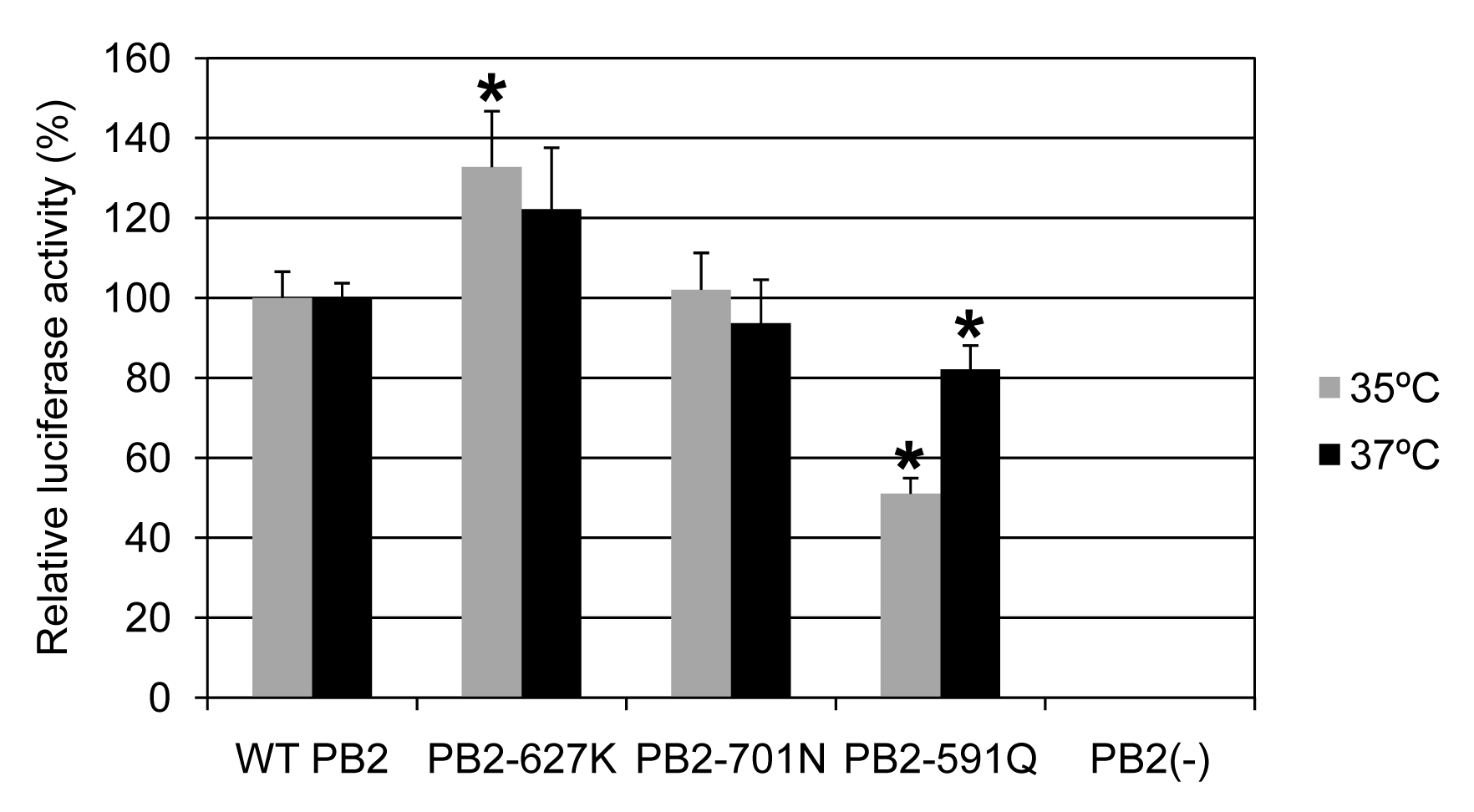

Supplement: Figure S2 — Polymerase activity of Cal04 PB2 variants in vitro. 293 cells were transfected with plasmids for the expression of a virus-like RNA encoding luciferase, and with plasmids encoding the Cal04 PB1, PA, NP, and wild-type or mutant PB2 proteins. Forty-eight hours later, luciferase activity was assessed. (0.10 MB TIF) [file ppat.1001034.s002.tif]

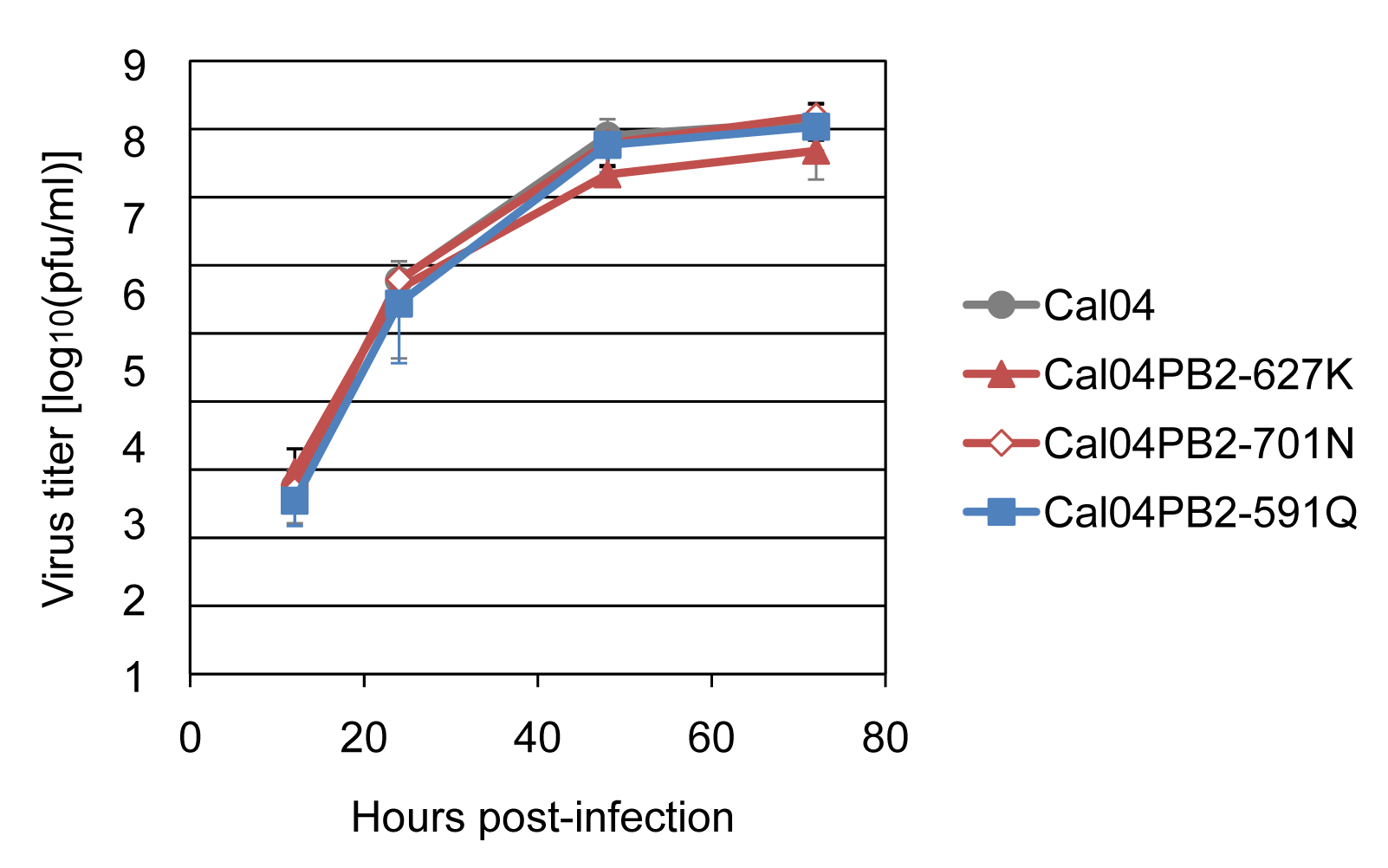

Supplement: Figure S3 — Growth curves of wild-type and mutant Cal04 viruses in MDCK cells. Cells were infected with wild-type or mutant Cal04 viruses at an m.o.i. of 0.001. At the indicated times post-infection, the virus titers in the cell culture supernatant were assessed by plaque assays. Experiments were carried out in triplicate. (0.10 MB TIF) [file ppat.1001034.s003.tif]

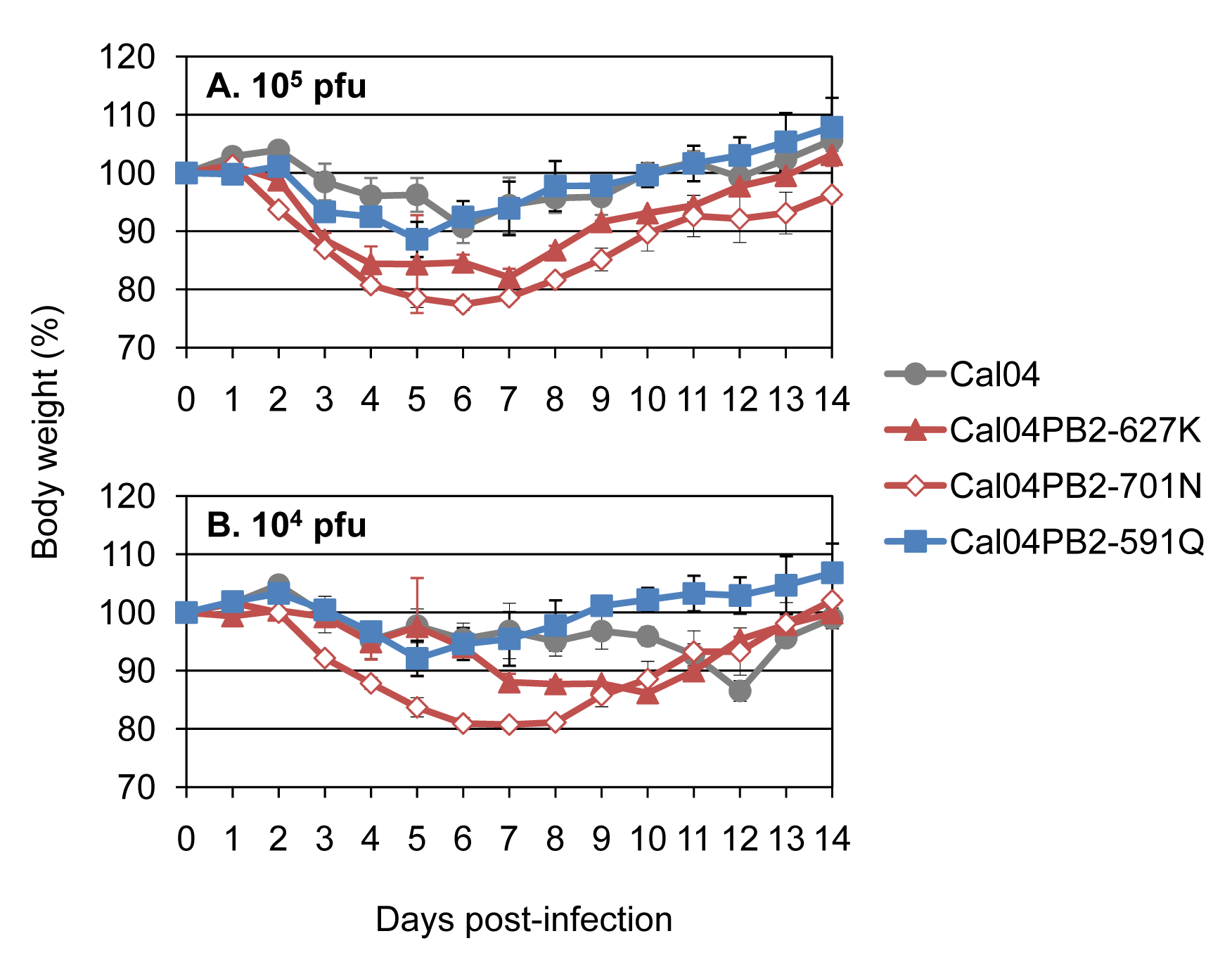

Supplement: Figure S4 — Virulence of wild-type and mutant Cal04 viruses in mice. BALB/c mice were infected with 105 PFU (A) or 104 PFU (B) of the indicated viruses and assessed daily for weight losses. For mice infected with the PB2-627K or PB2-701N variants, one mouse each had to be euthanized on day 5 or 6, respectively, due to body weight loss of more than 25% of the respective pre-infection body weight. (0.17 MB TIF) [file ppat.1001034.s004.tif]
