# Supplementary material for: Biological and Structural Characterization of a Host-Adapting Amino Acid in Influenza Virus
Source: PLoS Pathog. 2010 Aug 5;6(8):e1001034. doi: 10.1371/journal.ppat.1001034 (PMC2916879; doi:10.1371/journal.ppat.1001034)
Supplement: Table S1 — Biological features of PB2 variants in mice (0.03 MB DOC) [file ppat.1001034.s005.doc]

**Supplementary information**

**Supplementary Table 1. Biological features of PB2 variants in mice**

| Virus | Tissue tropism^a^ | | |
| --- | --- | --- | --- |
|  | Day post-infection | Virus titer (mean log10 PFU ± SD/g) in: | |
|  |  | Lungs | Nasal turbinates |
| Cal04 | 3 | 7.6±0.5 | 6.6±0.01 |
|  | 6 | 6.2±0.1 | 5.8±0.6 |
| Cal04PB2-627K | 3 | 7.3±0.1 | 6.6±0.03 |
|  | 6 | 5.9±0.1 | 5.4±0.3 |
| Cal04PB2-701N | 3 | 7.3±0.05 | 6.7±0.2 |
|  | 6 | 6.2±0.3 | 5.8±0.6 |
| Cal04PB2-591Q | 3 | 7.1±0.2 | 6.5±0.3 |
|  | 6 | 5.6±0.3 | 5.5±0.2 |

^a^Six week old BALB/c mice, anesthetized with isoflurane, were infected intranasally with 50 µl of virus (10^5^ PFU). Three mice from each infected group were sacrificed on day 3 and 6 post-infection for virus titration. None of the viruses tested was recovered from the spleens, kidneys, brains, colons, or livers of infected animals.
